# SINGLE-CELL AND SPATIAL OMICS FOR MAPPING CELLULAR SENESCENCE IN HEALTH, AGING AND DISEASE

**DOI:** 10.1093/geroni/igad104.1555

**Published:** 2023-12-21

**Authors:** Rong Fan

**Affiliations:** Yale University, New Haven, Connecticut, United States

## Abstract

NIH SenNet consortium aims to dissect the heterogeneity of senescent cells (SnCs) and map their impact on the microenvironment at a single cell resolution and in the spatial tissue context, which requires the implementation of an array of omics technologies to comprehensively identify, characterize, and spatially profile SnCs across tissues in humans and mice. These technologies are broadly categorized into two groups –single cell omics and spatial mapping. To achieve single cell resolution and overcome the scarcity of SnCs, high-throughput single-cell and single-nucleus transcriptomic techniques have become a mainstay tool for surveying tens of thousands of cells to identify transcriptional signatures in rare cell populations, enabling discovery of potential new SnC biomarkers. Novel single cell mass spectrometry methods are developed for unbiased discovery of proteomic signatures of SnCs. A hallmark of SnCs is the senescence-associated secretory phenotype (SASP), which requires the use of proteomics, secretomics, metabolomics and lipidomics, especially SASP-associated extracellular vesicles, for comprehensive characterization of SAPS. High resolution molecular and cellular imaging of gene expression (e.g., MERFISH) or protein markers (e.g., CODEX) is critical for the study of SnCs in the large-scale tissue context. NGS-based spatial omics sequencing is poised to bridge the gap to realize both genome scale and cellular resolution in mapping SnCs in tissue. Novel technologies such as Seq-Scope and Pixel-Seq developed within SenNet further enabled subcellular resolution. SenNet investigators also developed spatially resolved epigenome and multi-omics sequencing techniques to link transcriptional or proteomic phenotype of SnCs to epigenetic mechanism. Further integration with high-resolution imaging makes spatial omics the crucial linchpin in connecting mechanistic underpinnings and molecular signatures with morphological features and spatial distribution. All these are critical for the construction of a map of SnCs and associated niches in the native tissue environment implicated in human health, aging, and disease, which is one of the main goals of the SenNet consortium.

